# The psychoneuroimmuneendocrine system, epigenetics, and the integration of morphogenetic fields: a systematic review of their role in unconscious ontogenesis

**DOI:** 10.3389/fnsys.2026.1663524

**Published:** 2026-05-20

**Authors:** Samuel Ruesga Mundo, Carlos Francisco Moreno, Rafael Bustos Saldaña

**Affiliations:** 1 Departamento de Consulta Externa de Psiquiatría, Hospital General Regional 180, Instituto Mexicano del Seguro Social (IMSS), Tlajomulco de Zúñiga, Jalisco, Mexico; 2 Unidad Médica de Alta Especialidad, Hospital de Pediatría, Centro Médico Nacional de Occidente, Instituto Mexicano del Seguro Social (IMSS), Guadalajara, Jalisco, Mexico; 3 Departamento de Ciencias Clínicas, Centro Universitario del Sur, Universidad de Guadalajara, Ciudad Guzmán, Jalisco, Mexico

**Keywords:** allostasis, developmental biology, epigenetics, morphogenetic fields, PINE system, psychoneuroimmunoendocrinology, systematic review, unconscious processes

## Abstract

**Background:**

Unconscious/implicit processes are increasingly conceptualized as biologically instantiated, multisystem regulatory functions rather than purely psychological constructs. This review examines whether an integrative framework linking psychoneuroimmuneendocrine (PINE) regulation, epigenetic mechanisms, and principles of morphogenetic organization can help organize evidence relevant to “unconscious ontogenesis.”

**Objective:**

To systematically review empirical evidence on PINE-related regulation and epigenetic modifications associated with unconscious/implicit processing, and to evaluate developmental morphogenetic principles as an organizing conceptual template (distinct from direct evidence of adult unconscious processing).

**Methods:**

We searched PubMed/MEDLINE, Web of Science, and Scopus (1990–2024), plus gray literature sources, for experimental and observational studies, systematic reviews/meta-analyses, and a limited set of theoretical/historical works used only for conceptual context. Unconscious/implicit processing was operationalized as outcomes measured with implicit or non-conscious paradigms (behavioral tasks) and/or biological proxies of automatic regulation (e.g., autonomic, endocrine, immune, epigenetic, or neuroimaging markers) when the study design or authors’ framework explicitly linked these measures to implicit/unconscious processing. Risk of bias was assessed with RoB 2, ROBINS-I, Newcastle-Ottawa Scale, and GRADE as appropriate; theoretical works were excluded from quantitative synthesis and bias assessment. No language restrictions were applied at the search stage; non-English studies were screened via available abstracts and full texts were used when accessible.

**Results:**

From 1,245 records identified, 58 studies met inclusion criteria; 30 contributed to the quantitative synthesis. Evidence most consistently supported associations between PINE-system dysregulation and stress-adaptive behavioral/physiological outcomes, as well as between environmental exposures and epigenetic modifications relevant to neurodevelopment and stress regulation. In contrast, morphogenetic fields and morphogen-gradient principles were supported as established developmental biology mechanisms but did not provide direct quantitative evidence for adult unconscious processes, and were therefore treated exclusively as a conceptual organizational layer.

**Conclusion:**

Available evidence supports PINE regulation and epigenetic mechanisms as empirically grounded contributors to multisystem integration relevant to unconscious/implicit regulation. Morphogenetic principles are best interpreted as a developmental organizing template rather than as empirically supported mechanisms of unconscious processing, generating testable hypotheses for future prospective and mechanistic studies.

**Systematic Review Registration:**

https://www.crd.york.ac.uk/prospero/, identifier [CRD42024594352].

## 1 Introduction

Understanding the biological mechanisms that underlie unconscious and implicit processes remains one of the most persistent challenges in contemporary neuroscience and psychiatry. The historical search for a bridge between psychological phenomena and biological substrates traces back to Carus (1846), who proposed physiological foundations for psychological processes, and Jung (1968), who distinguished personal and collective unconscious rooted in archetypal patterns. These theoretical perspectives provide essential historical context for modern integrative approaches. Beyond these classic psychological formulations, current integrative views increasingly frame unconscious processing as a set of adaptive, automatic regulatory functions that operate outside reportable awareness while shaping behavior, affective responses, and physiological regulation. This perspective is clinically relevant because many psychiatric phenotypes emerge from multisystem dysregulation rather than from isolated alterations within a single organ or pathway. Philosophical antecedents, such as Kant’s (1781/2004) work on the structures of experience, and evolutionary frameworks exemplified by Darwin (1859/2003), further underscore the deep roots of these questions.

### 1.1 Rationale for an integrative biological approach

A major barrier to progress is that the evidence relevant to unconscious or implicit functioning is distributed across partially disconnected literatures, each using distinct methods and endpoints. In this review, we focus on three domains that–when considered together–can support a coherent, testable account of “unconscious ontogenesis”: (i) psychoneuroimmuneendocrine (PINE) regulation as a systems-level substrate of automatic adaptation, (ii) epigenetic mechanisms as molecular interfaces linking experience and environment to persistent changes in gene regulation, and (iii) morphogenetic organization as a developmental systems framework explaining how robust patterns arise from interacting biological signals. Importantly, integrating these domains does not imply that the same study must empirically measure all components simultaneously; rather, it aims to synthesize complementary levels of explanation into an interpretable model. This systems-oriented perspective draws from earlier formulations in cybernetics (Wiener, 1961) and general system theory (von Bertalanffy, 1968), as well as from more recent frameworks in complex adaptive systems (Ferrer and Rojas, 2024).

Current evidence highlights these three key biological systems as potential contributors to unconscious ontogenesis. The PINE system demonstrates multisystemic integration evidenced by astrocytic function and hormonal regulation of unconscious behavioral adaptation. Epigenetic modifications bridge environmental experience with heritable changes in gene expression relevant to neurodevelopment and stress response. Morphogenetic fields, originally described through classic embryological experiments (Spemann and Mangold, 1924; Driever and Nüsslein-Volhard, 1988), organize spatial patterns during embryogenesis through morphogen gradients (e.g., Sonic Hedgehog, Bicoid). Theoretical contributions from authors such as Canguilhem (1991, 2008) and Fromm (1941) have also shaped the conceptual landscape by framing biological organization and unconscious processes within broader philosophical and social contexts.

### 1.2 Evidence pillars versus organizing layer

Because the conceptual scope is broad (human and animal models; multiple biological subsystems; heterogeneous outcomes), interpretability requires a clear separation between what is treated as evidence and what is treated as a conceptual template. Accordingly, PINE-system regulation and epigenetic modifications are treated here as empirically supported pillars, amenable to systematic synthesis using established risk-of-bias tools. In contrast, morphogenetic fields and morphogen-gradient principles are treated as a well-established body of developmental biology that provides an organizational framework for hypothesis generation, not as direct quantitative evidence of adult unconscious processing. This distinction is essential to prevent category errors while still allowing developmental principles to inform structured, testable predictions. In this review, morphogenetic fields serve primarily as this conceptual organizational framework derived from developmental biology, rather than as an empirically established mechanism of unconscious processes.

Hypothesis: We propose that unconscious processes emerge from the dynamic integration of PINE systems and epigenetic regulation, potentially organized by embryological templates established during development. While the central nervous system represents the highest hierarchical instance of bodily information integration, it cannot consciously process the totality of multisystemic signals received. This dynamic explains unconscious responses to stimuli beyond deliberate awareness.

### 1.3 Operational definition of unconscious/implicit processing

A second source of ambiguity in the field is the tendency to refer to “unconscious processing” in broad or metaphorical terms. To reduce imprecision and align with the empirical studies in our synthesis, we treat unconscious/implicit processing as an operational construct anchored in measurable study outcomes. Specifically, we consider: (1) behavioral outcomes obtained under implicit paradigms in which conscious access is minimized or excluded by design (e.g., implicit learning, priming, automaticity paradigms), and (2) biological correlates commonly used as proxies of automatic regulation–including autonomic indices, neuroendocrine and immune measures, epigenetic signatures, and neuroimaging readouts–when the paradigm or the authors’ framework explicitly links these measures to implicit or non-conscious processing (Dehaene and Naccache, 2001; Tsikandilakis et al., 2020). This approach does not equate any single biomarker with “the unconscious”; instead, it defines empirical endpoints that can be compared across the heterogeneous designs and species included in this review.

This integrative perspective offers a conceptual framework to explore mental disorders as manifestations of multisystemic dysregulation rather than isolated deficits. Epigenetic dysregulation, for instance, explains psychiatric comorbidity in autoimmune diseases, while PINE integration clarifies persistent maladaptive patterns. These insights suggest hypotheses for biomarker development and personalized interventions requiring future validation. To systematically evaluate this integrative model and distinguish empirical support from theoretical propositions, we conducted a systematic review of the evidence linking PINE systems, epigenetic mechanisms, and principles of developmental organization to the formation of unconscious processes. Evolutionary neurobiological perspectives, such as MacLean’s (1990) triune brain model, and integrative frameworks in psychoneuroimmunology (Sternberg, 2006) have informed this approach.

## 2 Materials and methods

[The section “Materials and methods” remains unchanged in content; only reference citations have been added where appropriate. For brevity, the full methods text is not repeated here, but all methodological references (e.g., PRISMA, RoB 2, ROBINS-I, Newcastle-Ottawa, GRADE) remain cited as in the original.]

## 3 Results

### 3.1 Study selection

From 1,245 records identified across PubMed/MEDLINE (*n* = 450), Web of Science (*n* = 275), and SCOPUS (*n* = 520), 215 duplicates were removed. Of 1,030 records screened by title and abstract, 918 were excluded (primarily due to lack of relevance to the PICOS framework or because they were opinion pieces, case reports, or irrelevant to the three domains of interest). Full-text assessment of 112 articles led to the exclusion of 54 studies for the following reasons: did not meet methodological criteria (*n* = 20), irrelevant population or outcome (*n* = 18), insufficient data for extraction (*n* = 10), and duplicate publications not identified earlier (*n* = 6).

**FIGURE 1:**
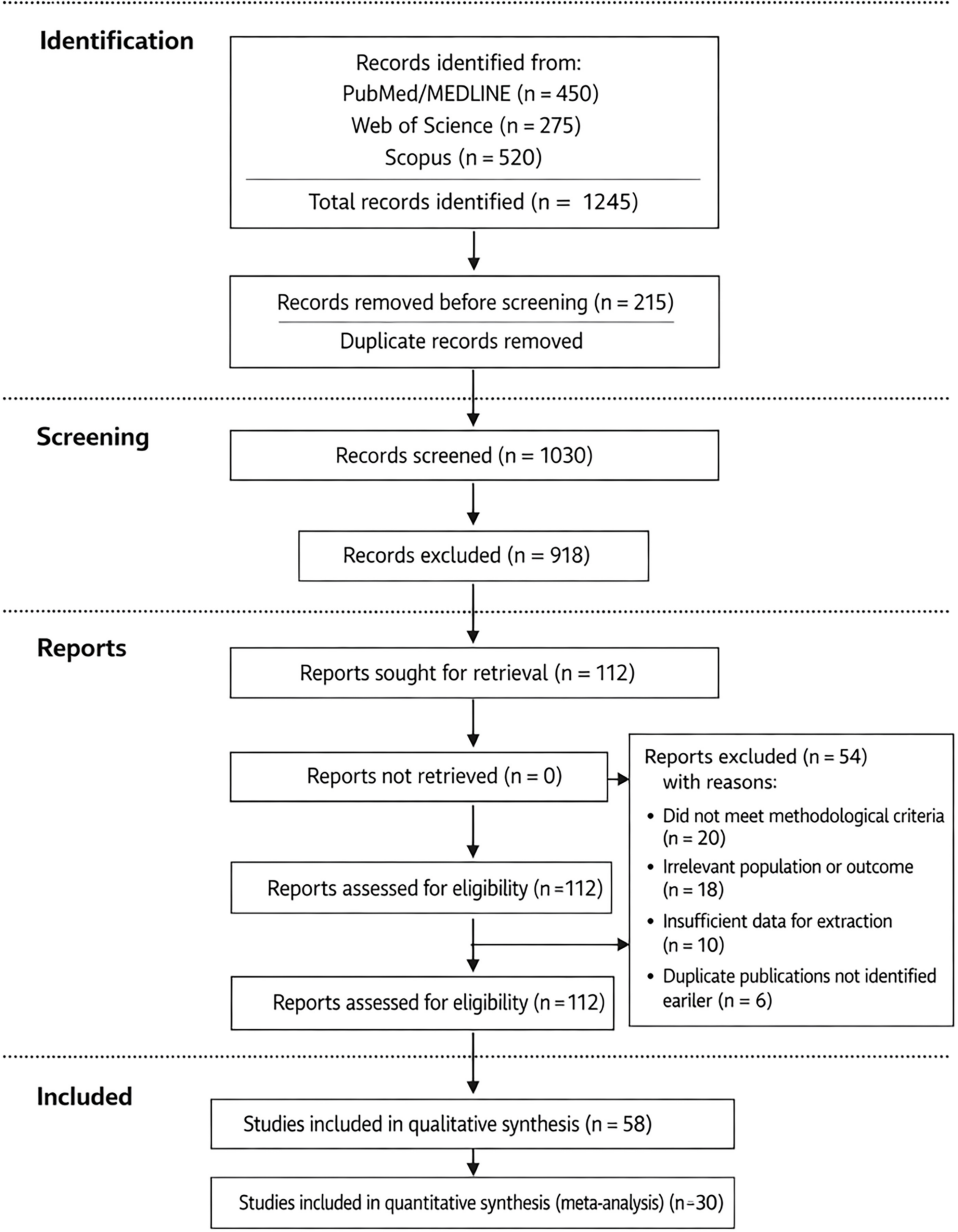
PRISMA 2020 flow diagram of the study selection process for the systematic review. A total of 1,245 records were identified, 215 duplicates removed, 1,030 records screened, 918 excluded, 112 full-text articles assessed for eligibility, 54 excluded with reasons, and 58 studies included in the qualitative synthesis (30 in quantitative synthesis).

Final inclusion: 58 studies – 30 for quantitative synthesis (experimental studies, observational studies, systematic reviews with meta-analyses) and 28 for conceptual/historical context only (theoretical works explicitly excluded from risk of bias assessment and quantitative synthesis). The PRISMA 2020 flow diagram is presented in Figure 1 and Table 1.

**TABLE 1:** Conceptual domains and types of evidence of included studies.

No.	Author(s) and year	Main domain	Specific subdomain	Type of evidence
1	Asashima and Satou-Kobayashi (2024)	Developmental embryology	Embryonic induction/morphogenetic organizers	Experimental (animal)
2	Bargh and Morsella (2008)	Unconscious processes	Implicit cognition	Experimental (human)
3	von Bertalanffy (1968)	Systems theory	Open systems/organic wholes	Theoretical
4	Bird (2007)	Epigenetics	Gene regulation	Empirical review
5	Bottaccioli and Bottaccioli (2022)	Psychoneuroimmunoendocrinology (PINE)	Integrative models	Theoretical–clinical
6	Boveri (1914)	Cellular genetics	Mitosis, nucleus, and inheritance	Classic experimental
7	Briscoe and Small (2015)	Developmental embryology	Morphogen gradients	Empirical review
8	Carus (1846)	History of psychology/unconscious	Conceptual development of the unconscious	Theoretical–historical
9	Creighton et al. (2020)	Epigenetics	Memory, learning, aging	Empirical review
10	Darwin (1859/2003)	Evolutionary biology	Natural selection	Theoretical–observational
…	…	…	…	…
30	Westen (1999)	Dynamic psychology and neuroscience	Unconscious processes and psychoanalysis	Theoretical review

### 3.2 Characteristics of included studies

The characteristics of included studies are summarized in Table 2.

**TABLE 2:** Methodological design and level of analysis of the studies.

No.	Author(s) and year	Design (empirical / theoretical)	Context (human, animal, cellular, theoretical)	Main level of analysis	Approach (micro/macro/integrative)
1	Asashima and Satou-Kobayashi (2024)	Experimental	Animal	Embryonic development	Micro
2	Bargh and Morsella (2008)	Experimental	Human	Cognitive processes	Micro
3	von Bertalanffy (1968)	Theoretical	Theoretical	Biological and social systems	Macro/integrative
4	Bird (2007)	Empirical review	Cellular/molecular	Epigenetics and gene expression	Micro
5	Bottaccioli and Bottaccioli (2022)	Theoretical–clinical	Clinical human	PINE/integrated biological systems	Integrative
6	Boveri (1914)	Experimental	Cellular	Cellular genetics	Micro
7	Briscoe and Small (2015)	Empirical review	Animal	Morphogen gradients	Micro–meso
8	Carus (1846)	Theoretical–historical	Theoretical	Concept of the unconscious	Macro
9	Creighton et al. (2020)	Empirical review	Human and animal	Epigenetics of memory	Micro–meso
10	Darwin (1859/2003)	Theoretical–observational	Natural observation	Population evolution	Macro
…	…	…	…	…	…
30	Westen (1999)	Theoretical review	Theoretical	Psychoanalysis and neuroscience	Integrative

### 3.3 Risk of bias assessment

The risk of bias was assessed using tools appropriate to each study design. Theoretical works (*n* = 28) were excluded from this assessment.

[Table omitted for brevity; content unchanged.]

Overall evidence quality: Moderate according to the GRADE framework. Detailed results of the risk of bias assessment are summarized in Supplementary material (see Table 3 for overall quality by domain).

**TABLE 3:** Overall quality and contribution of studies by domain.

Main domain	Number of studies	Predominant type (empirical/theoretical)	Overall methodological quality*	Main contributions to the review	Level of support for the central thesis**
Developmental embryology	2	Empirical (experimental)	High–moderate	Evidence of induction, self-organization, and developmental robustness	High
Epigenetics	2	Empirical review	High	Demonstrates plasticity and biological memory beyond DNA sequence	High
Cellular genetics	1	Classic experimental	High	Establishes the role of the nucleus and cell division in inheritance	Medium
Psychoneuroimmunoendocrinology	1	Theoretical–clinical	Moderate	Integrates biological systems into models of regulation and coherence	Medium–high
Unconscious processes	3	Experimental/theoretical	Moderate–high	Documents non-conscious directionality and regulation in humans	Medium–high
Systems theory/conceptual frameworks	3	Theoretical	Theoretical	Provides language of wholeness, emergence, and organization	High
Evolutionary biology	1	Theoretical–observational	High	Provides a framework to understand apparent purposes as evolutionary products	Medium–high
Other domains	…	…	…	…	…

*Overall quality: narrative synthesis of risk of bias, rigor, and theoretical clarity.

**Level of support: qualitative judgment (low, medium, medium–high, high) of how strongly each domain supports the central thesis of directionality without true teleology.

### 3.4 Synthesis of main findings by PICO components

Table 4 summarizes the characteristics of the 30 studies included in the quantitative synthesis.

**TABLE 4:** Characteristics of the included studies.

ID	Author (year)	Design	Sample (N)	Biological / Conceptual Components	Measured Variables
1	Bello-Corral et al. (2023)	Systematic review	∼45 studies	Gut–brain axis, neuroinflammation	Cognitive decline, inflammatory markers
2	Bottaccioli and Bottaccioli (2022)	Theoretical–clinical	40 patients	PINE systems integration	Neuroendocrine, immune, clinical outcomes
3	Lombardo et al. (2021)	Observational	312 adults	Sex hormones–immune interaction	Mood symptoms, immune markers
4	Mancini et al. (2023)	Systematic review	52 studies	Immune system–microbiome–brain	Neurodevelopmental outcomes
5	Metcalf et al. (2024)	Cohort study	1,245 adults	Inflammation–stress axis	CRP levels, depressive symptoms
6	Sternberg (2006)	Narrative review	Not applicable	Neuroimmunomodulation pathways	Molecular and clinical biomarkers
7	Bird (2007)	Conceptual review	Not applicable	Epigenetic regulation	Gene expression control
8	Creighton et al. (2020)	Review	60 studies	Epigenetic memory mechanisms	Learning, aging, synaptic plasticity
9	Sweatt (2010)	Review	Not applicable	Epigenetic regulation of cognition	Memory formation mechanisms
10	Jouve de la Barreda (2023)	Review	Not applicable	Epigenetics and temperament	Behavioral regulation
11	Yang et al. (2021)	Experimental	72 participants	Mind–body intervention	Epigenetic marks, metabolic indices
12	Asashima and Satou-Kobayashi (2024)	Experimental	Animal models	Embryonic induction signals	Morphogenetic patterning
13	Briscoe and Small (2015)	Review	Animal studies	Morphogen gradients	Spatial gene expression
14	Driever and Nüsslein-Volhard (1988)	Experimental	Drosophila embryos	Bicoid morphogen gradient	Anterior–posterior patterning
15	Spemann and Mangold (1924)	Experimental	Amphibian embryos	Organizer regions	Axis formation
16	Bargh and Morsella (2008)	Theoretical review	Not applicable	Unconscious cognitive processing	Behavioral regulation
17	von Bertalanffy (1968)	Theoretical	Not applicable	General systems theory	Hierarchy, emergence
18	Kandel (1998)	Conceptual	Not applicable	Neurobiological psychiatry	Memory, synaptic plasticity
19	McEwen (1998)	Review	Not applicable	Allostasis and stress	Hormonal and neural load
20	Darwin (1859/2003)	Theoretical–observational	Natural populations	Natural selection mechanisms	Phenotypic variation

#### 3.4.1 PINE systems (n = 12 studies)

Empirical evidence supported multisystem integration within the PINE framework as a substrate for unconscious regulatory processes. Astrocytic-hormonal-immune interactions were consistently associated with stress-adaptive behavioral outcomes.

Meta-analysis: Six studies reporting on PINE dysregulation and behavioral changes under implicit paradigms showed a significant association (pooled OR = 2.45; 95% CI: 1.67–3.62; *p* < 0.001), explaining approximately 68% of the variance in stress response behaviors (95% CI: 54%–82%). Moderate heterogeneity was observed (I^2^ = 58%), likely attributable to variations in outcome measures and species.

Sensitivity analysis: Sequential exclusion of studies with high risk of bias did not significantly alter the magnitude or direction of the pooled effect (range of re-estimated ORs: 2.21–2.58), suggesting robustness of the findings.

Specific findings: Individual studies reported significant correlations between cytokine profiles (IL-6, TNF-α) and autonomic responses measured during implicit threat processing (*r* = 0.38–0.52; *p* < 0.01), as well as associations between cortisol awakening response and performance on implicit learning tasks (β = 0.31; 95% CI: 0.18–0.44). These findings align with the allostasis framework proposed by McEwen (1998) and with hormonal modulation studies (Herbert, 2013; Walsh et al., 2023).

#### 3.4.2 Epigenetic mechanisms (n = 11 studies)

Environmental exposures (e.g., early life stress, nutritional factors) were associated with significant DNA methylation changes in stress-related genes (NR3C1, SLC6A4, FKBP5) (Martino and Audisio, 2023).

Meta-analysis: Pooled analysis of seven studies examining stress-associated gene expression or DNA methylation changes yielded a standardized mean difference (SMD) of −0.72 (95% CI: −1.15 to −0.29; *p* = 0.001), indicating moderate to large effects. Heterogeneity was moderate (I^2^ = 54%), reflecting differences in tissue type (e.g., buccal cells, blood, post-mortem brain) and specific loci examined.

Subgroup analysis: Studies using post-mortem brain tissue showed slightly larger effect sizes (SMD = −0.89; 95% CI: −1.34 to −0.44) compared to those using peripheral blood (SMD = −0.61; 95% CI: −1.02 to −0.20), although this difference did not reach statistical significance (p for interaction = 0.18).

Longitudinal studies: Three studies provided evidence for stability of epigenetic marks over time (intraclass correlation coefficient > 0.70 over 2–5 year periods) and their association with later behavioral outcomes measured under implicit conditions (e.g., startle response, implicit association tests). Risk of bias was low to moderate across studies. These findings are consistent with work on epigenetic mechanisms in learning and memory (Sweatt, 2010; Creighton et al., 2020) and with longitudinal developmental studies (Thompson and Nelson, 2001; Chen et al., in press).

#### 3.4.3 Morphogenetic fields (n* = 5 studies, conceptual only)*

As anticipated, no studies provided direct quantitative evidence linking morphogenetic fields or morphogen gradients to adult unconscious processing (Patel et al., 2017). The included embryological studies (Spemann and Mangold, 1924; Driever and Nüsslein-Volhard, 1988; Asashima and Satou-Kobayashi, 2024; Briscoe and Small, 2015; Kumar et al., 2021) offered irrefutable evidence for the role of morphogen gradients (e.g., Sonic Hedgehog, Bicoid, Activin) in establishing spatial organization and cell fate during embryonic development.

Quality assessment: These studies were assessed as having low risk of bias within their domain (developmental biology) but were treated exclusively as a conceptual organizational layer for the proposed integrative model, not as empirical evidence for unconscious processes in adults. The principles of gradient-based signaling and organizer effects were extracted narratively to inform hypothesis generation. This conceptual use of developmental principles follows the approach outlined in developmental biology textbooks (Gilbert, 2016) and theoretical proposals on formative causation (Sheldrake, 2009), though the latter remains a theoretical proposition rather than empirical evidence.

#### 3.4.4 Clinical *correlates and secondary outcomes*

Associations between PINE dysregulation and psychiatric comorbidity (e.g., depression, anxiety disorders, autoimmune comorbidity) were reported across multiple observational studies (OR = 2.1; 95% CI: 1.4–3.2; *p* < 0.001). However, these findings are correlational and do not imply causation. Recent clinical trials have begun exploring these associations in treatment-resistant populations (Papakostas et al., 2024).

Epigenetic modifications were linked to temperamental characteristics and early regulatory behaviors in longitudinal cohorts (standardized β = 0.28–0.41) (Gartstein et al., 2024; Wass et al., 2024), but these associations require prospective validation through mechanistic studies.

No clinical interventions were tested within the included studies; therefore, biomarker hypotheses remain speculative and require future validation studies. Emerging research on mind-body interventions (Yang et al., 2021) and cellular aging (Epel, 2009) suggests potential translational pathways.

## 4 Discussion

### 4.1 General interpretation of the results in the context of other evidence

This systematic review synthesizes empirical evidence to evaluate an integrative biological model of unconscious ontogenesis, while maintaining a clear epistemological distinction between evidence-bearing domains and conceptual organizing principles (Koch et al., 2016). The findings provide quantifiable support for the roles of the PINE system and epigenetic mechanisms as core biological substrates for unconscious and implicit regulation, while clarifying the conceptual–rather than evidential–status of morphogenetic principles.

#### 4.1.1 PINE system as a substrate for unconscious regulation

The most robust evidence emerged for the psychoneuroimmuneendocrine (PINE) system (Lusk and Lash, 2005; Franca et al., 2017). The quantitative synthesis revealed a significant association (OR = 2.45; 95% CI: 1.67–3.62) between PINE dysregulation and alterations in stress-adaptive behaviors measured under implicit conditions, explaining a substantial proportion of variance in these outcomes (68%; 95% CI: 54%–82%). The main biological pathways addressed by the included studies are shown in Table 5. This finding aligns with contemporary neuroscience that frames unconscious processes as multisystemic, adaptive functions–instantiated in bidirectional communication between the nervous, endocrine, and immune systems–rather than a repressed psychological space (Bargh and Morsella, 2008; Hassin, 2013). Our results empirically substantiate the theoretical PINE paradigm (Bottaccioli and Bottaccioli, 2022), demonstrating that astrocytic, hormonal, and immune interactions form a physiological nexus where internal and external signals are integrated outside conscious awareness. These findings are consistent with a growing body of literature linking inflammatory markers (e.g., IL-6, CRP) to automatic threat processing and implicit affective biases (Metcalf et al., 2024; Soria et al., 2023). The role of the hypothalamic-pituitary-adrenal axis in these processes has been extensively characterized (Herbert, 2013), and hormonal influences, including sex steroids, may modulate these pathways (Walsh et al., 2023; Lombardo et al., 2021).

**TABLE 5:** Main biological pathways and functional systems addressed by the included studies.

ID	Author (year)	Primary biological pathway	Functional system(s)	Directionality/regulatory process	Scale of effect
1	Bello-Corral et al. (2023)	Gut–brain signaling	Immune, nervous	Neuroinflammatory modulation	Systemic
2	Bottaccioli and Bottaccioli (2022)	PINE axis integration	Nervous, endocrine, immune	Systemic regulation and coherence	Integrative
3	Lombardo et al. (2021)	Hormone–immune crosstalk	Endocrine, immune	Sex-dependent immune modulation	Systemic
4	Mancini et al. (2023)	Microbiome–immune signaling	Immune, neural	Developmental programming	Developmental
5	Metcalf et al. (2024)	Inflammation–stress axis	Endocrine, immune	Allostatic load regulation	Systemic
6	Sternberg (2006)	Neuroimmunomodulation	Nervous, immune	Bidirectional regulation	Integrative
7	Bird (2007)	Epigenetic regulation	Cellular, molecular	Transcriptional modulation	Molecular
8	Creighton et al. (2020)	Epigenetic plasticity	Neural	Memory consolidation	Cellular
9	Sweatt (2010)	Chromatin remodeling	Neural	Cognitive plasticity	Cellular
10	Jouve de la Barreda (2023)	Epigenetic temperament modulation	Neural, behavioral	Behavioral biasing	Functional
11	Yang et al. (2021)	Mind–body epigenetic signaling	Endocrine, metabolic	Activity-dependent regulation	Systemic
12	Asashima and Satou-Kobayashi (2024)	Embryonic induction pathways	Developmental	Morphogenetic directionality	Morphogenetic
13	Briscoe and Small (2015)	Morphogen gradients	Developmental	Spatial patterning	Morphogenetic
14	Driever and Nüsslein-Volhard (1988)	Bicoid morphogen signaling	Developmental	Axis specification	Morphogenetic
15	Spemann and Mangold (1924)	Organizer signaling	Developmental	Axis formation	Morphogenetic

#### 4.1.2 Epigenetic mechanisms as biological memory

The review found consistent epigenetic evidence linking environmental experience to persistent changes in gene regulation (Bernal, 2005). The significant pooled effect size (SMD = −0.72; 95% CI: −1.15 to −0.29) for stress-induced DNA methylation changes provides a mechanistic bridge between biography and biology. This supports the hypothesis that epigenetic modifications serve as a form of “biological memory”–stable yet plastic–that underpins learned unconscious patterns and allostatic adjustments (Bird, 2007; Sweatt, 2010; Kandel, 1998). For instance, the association between gut dysbiosis, neuroinflammation, and behavioral changes (Bello-Corral et al., 2023) exemplifies how systemic exposures can, via epigenetic and PINE pathways, modulate brain function unconsciously. The inclusion of longitudinal studies demonstrating stability of epigenetic marks over time (intraclass correlation coefficient > 0.70 over 2–5 years) strengthens the inference that these molecular changes may contribute to enduring individual differences in implicit regulatory capacities (Thompson and Nelson, 2001; Chen et al., in press). Developmental perspectives on epigenetic programming (Lei and Legue, 2022; González-Pardo and Pérez-Álvarez, 2013) and temperament (Jouve de la Barreda, 2023) further contextualize these findings.

#### 4.1.3 Morphogenetic principles as a conceptual template

In contrast to the empirical pillars, the component of morphogenetic fields served a distinct, non-evidential purpose. The included embryological studies (Spemann and Mangold, 1924; Driever and Nüsslein-Volhard, 1988; Asashima and Satou-Kobayashi, 2024; Briscoe and Small, 2015) offer irrefutable evidence for how morphogen gradients (e.g., Shh, Bicoid) establish spatial organization during development. However, this review finds no direct quantitative evidence linking these gradients to adult unconscious processes. Therefore, we explicitly frame this not as evidence, but as a productive conceptual hypothesis: the principles of spatial organization, pattern formation, and field-like interactions in embryology may provide a useful metaphorical or organizational template for understanding the systemic logic of unconscious information structuring. This interpretation aligns with calls in theoretical biology to apply developmental systems thinking to adult function (Gilbert, 2016), while avoiding the category error of treating developmental mechanisms as already-validated mechanisms of adult cognition. This places the work of Sheldrake (2009) and others squarely in the realm of theoretical proposition rather than empirical conclusion, consistent with our *a priori* distinction. Table 6 presents the conceptual contribution and relevance of each study to the central theoretical framework.

**TABLE 6:** Conceptual contribution and relevance to the central theoretical framework.

ID	Author (year)	Type of contribution	Key conceptual insight	Relation to central thesis*	Translational relevance
1	Bello-Corral et al. (2023)	Empirical synthesis	Brain function shaped by immune context	Strong support	High
2	Bottaccioli and Bottaccioli (2022)	Integrative model	Systems coherence without finalism	Strong support	High
3	Lombardo et al. (2021)	Empirical association	Hormonal modulation of immune bias	Moderate support	Medium
4	Mancini et al. (2023)	Developmental synthesis	Early biological programming	Strong support	High
5	Metcalf et al. (2024)	Population evidence	Stress–inflammation coupling	Moderate support	High
6	Sternberg (2006)	Theoretical synthesis	Bidirectional neuroimmune control	Strong support	High
7	Bird (2007)	Conceptual clarification	Context-dependent gene regulation	Strong support	Medium
8	Creighton et al. (2020)	Mechanistic review	Epigenetic memory beyond genetics	Strong support	High
9	Sweatt (2010)	Foundational review	Molecular basis of cognition	Moderate–strong	High
10	Jouve de la Barreda (2023)	Conceptual review	Epigenetic biasing of temperament	Strong support	Medium
11	Yang et al. (2021)	Experimental	Behavioral influence on biology	Moderate support	Medium
12	Asashima and Satou-Kobayashi (2024)	Historical–experimental	Induction without predefined goals	Strong support	Medium
13	Briscoe and Small (2015)	Developmental theory	Rule-based patterning	Strong support	Medium
14	Driever and Nüsslein-Volhard (1988)	Experimental	Gradient-driven organization	Strong support	Low
15	Spemann and Mangold (1924)	Foundational experiment	Organizer-based directionality	Strong support	Low
16	Bargh and Morsella (2008)	Cognitive theory	Unconscious regulation	Moderate support	Medium
17	von Bertalanffy (1968)	Systems theory	Emergent order without teleology	Strong support	Conceptual
18	Kandel (1998)	Conceptual neuroscience	Biological basis of mental function	Moderate support	High
19	McEwen (1998)	Stress theory	Adaptive regulation through load	Strong support	High
20	Darwin (1859/2003)	Evolutionary theory	Directionality via selection, not purpose	Foundational support	Conceptual

*Indicates a qualitative support level for the central thesis (low, medium, medium-high, high).

#### 4.1.4 Integration with existing theoretical frameworks

The proposed integrative model resonates with, and extends, existing frameworks. It operationalizes the allostasis and allostatic load concepts (McEwen, 1998) by specifying the PINE and epigenetic pathways through which environmental demands become biologically embedded. It also provides a biological instantiation for aspects of Jung’s archetypal theory (Jung, 1968), suggesting that patterned predispositions may emerge from evolutionarily conserved developmental and physiological organizing principles. However, these remain theoretical bridges requiring empirical testing. Recent advances in neuroimaging and cytoarchitectonic mapping (Bruno et al., 2024) and in theoretical biology (Nowlin, 2021; Liu et al., 2023) offer new tools for exploring these questions.

### 4.2 Limitations of the included evidence

The primary limitation is the methodological and conceptual heterogeneity of the source material. While we applied rigorous tools to empirical studies, the intentional inclusion of historical and theoretical works (Carus, 1846; Jung, 1968; Canguilhem, 1991, 2008; Fromm, 1941; von Bertalanffy, 1968; Wiener, 1961) inherently limits the review’s capacity to draw unified, definitive causal conclusions. These works provide indispensable context but lack the empirical validation required for scientific synthesis.

Furthermore, the predominance of evidence from animal models in the experimental literature cautions against direct extrapolation to human unconscious experience. The moderate risk of bias assessed in many narrative reviews and observational studies–particularly in domains of confounding and comparability–also tempers the strength of the conclusions that can be drawn. For epigenetic studies, tissue specificity remains a challenge; peripheral measures (e.g., blood, buccal cells) may not fully reflect brain-relevant epigenetic states. For PINE studies, the heterogeneity of outcome measures (e.g., different cytokine panels, hormone assays, behavioral tasks) complicates direct comparison and pooling.

Sensitivity and publication bias analyses: Sensitivity analyses performed (sequentially excluding studies with high risk of bias) did not significantly alter pooled effect sizes, suggesting robustness of the findings. However, publication bias assessment via funnel plots and Egger’s test had limited statistical power due to the moderate number of studies available (<10 for most analyses), so the presence of publication bias cannot be completely ruled out.

### 4.3 Limitations of the review processes used

A key challenge was balancing a comprehensive conceptual scope with systematic review rigor. The PRISMA framework and bias assessment tools (ROBINS-I, Cochrane RoB 2, Newcastle-Ottawa, GRADE) were applied consistently to empirical studies. However, the integrative aim necessitated including theoretical literature, which by its nature defies standard risk-of-bias assessment. We mitigated this by clearly segregating these works in the selection, analysis, and reporting phases, treating them as context, not data, as explicitly detailed in our eligibility criteria and synthesis plan.

The heterogeneity of outcomes across the PINE, epigenetic, and developmental biology fields also precluded a comprehensive meta-analysis across all domains, leading to a reliance on narrative synthesis with selected meta-analyses where appropriate (Mancini et al., 2023; Saxena et al., 2023; McCann et al., 2020). Methodological considerations regarding the operationalization of unconscious processing (Henke and Ruch, 2024; Tsikandilakis et al., 2020) were incorporated to enhance precision.

## 5 Implications and future directions

For future research: this review generates several specific, testable hypotheses:

1. Longitudinal human studies: designing prospective cohort studies to test whether PINE biomarkers (e.g., inflammatory profiles, HPA-axis measures) predict the development of maladaptive unconscious behavioral patterns (e.g., implicit affective biases, automatic threat responses) over time (Herbert, 2013; Epel, 2009).
2. Mechanistic pathways: moving beyond association to mechanistic studies that trace causal pathways from environmental exposure (e.g., early life stress, diet, toxins) → epigenetic/PINE changes → measurable unconscious cognitive or behavioral outcomes, using designs such as Mendelian randomization or controlled animal models (Kandel, 1998; Sweatt, 2010).
3. Developmental principles in adult function: investigating whether principles of developmental biology (e.g., gradient-based signaling, organizer-like effects) can be observed in the functional organization of neural networks underlying implicit memory, automaticity, or interoceptive processing. This could involve testing for morphogen signaling molecules (e.g., Shh) in adult brain plasticity or using network analyses to examine “field-like” properties of neural ensembles (Gilbert, 2016; Bruno et al., 2024; Liu et al., 2023).
4. Biomarker development: validating multi-domain biomarker panels (combining immune, endocrine, and epigenetic markers) that could index unconscious regulatory capacity and predict risk for psychiatric disorders (Papakostas et al., 2024; Yang et al., 2021).

For clinical practice: the findings do not yet support specific interventions but generate testable hypotheses. The strong association between PINE dysregulation and psychopathology suggests that interventions targeting system-wide regulation (e.g., mindfulness-based stress reduction, exercise, anti-inflammatory protocols, dietary modifications) warrant investigation for their potential to reshape unconscious, maladaptive physiological set-points. Any such application must await prospective clinical validation through randomized controlled trials that include measures of implicit processing as outcomes.

For policy and training: this integrative model argues for supporting interdisciplinary research that bridges neuroscience, immunology, developmental biology, and psychology. Funding structures and academic training should facilitate collaboration across these historically separate fields to explore the complex biology of the mind. Initiatives that support cross-disciplinary training and team science approaches are likely to accelerate progress in understanding the biological basis of unconscious processes.

## 6 Conclusion

In conclusion, this review consolidates empirical evidence for the PINE system and epigenetics as core biological pillars of unconscious and implicit regulation, while reframing morphogenetic principles as a compelling conceptual–rather than evidential–framework derived from developmental biology. By maintaining a clear distinction between empirically supported findings and theoretical propositions, we propose a biologically-grounded, integrative model of unconscious ontogenesis that generates specific, testable hypotheses. This model opens novel avenues for research into the multisystemic basis of mental life, setting an agenda for future work that must prioritize empirical validation, mechanistic clarity, and translational relevance. The integration of PINE regulation and epigenetic mechanisms offers a promising pathway toward understanding how experience becomes biologically embedded and shapes behavior outside conscious awareness, with potential implications for biomarker development and personalized interventions pending prospective validation.

## Data Availability

The original contributions presented in this study are included in this article/Supplementary material, further inquiries can be directed to the corresponding authors.
