# Supplementary material for: The psychoneuroimmuneendocrine system, epigenetics, and the integration of morphogenetic fields: a systematic review of their role in unconscious ontogenesis
Source: Front Syst Neurosci. 2026 May 20;20:1663524. doi: 10.3389/fnsys.2026.1663524 (PMC13230023; doi:10.3389/fnsys.2026.1663524)
Supplement: Supplementary file 1 [file Data_Sheet_1.pdf]

Tabla: Descripción de los artículos seleccionados para la revisión sistemática

Nº	Autor y Año	Título y Nº de Participantes	Objetivo y Calidad Metodológica	Resultados Principales	Conclusión
1	Asashima & Satou-Kobayashi (2024)*	Inducción embrionaria en modelos animales (n=50)	Evaluar mecanismos moleculares; calidad moderada	Evolución de la embriología molecular descrita	Confirma la importancia de la inducción embrionaria
2	Bargh & Morsella (2008)	Procesos inconscientes en humanos (n=120)	Analizar funciones cognitivas inconscientes; alta calidad	El inconsciente regula funciones cognitivas	Respalda la influencia del inconsciente en la cognición
3	Bertalanffy (1968)	Teoría de sistemas (no aplica)	Integrar sistemas biológicos y sociales; calidad teórica	Sistemas integrados biológicos y sociales	Fundamenta bases teóricas para estudios interdisciplinarios
4	Bird (2007)	Mecanismos epigenéticos y regulación génica (n=30)	Revisar mecanismos epigenéticos; calidad alta	Identificación de mecanismos epigenéticos	Epigenética clave en regulación génica
5	Bottaccioli & Bottaccioli (2022)	Paradigma neuroendocrinoinmunológico (n=40)	Explorar sistema PINE; calidad moderada	Paradigma integrativo neuroendocrinoinmunológico	Refuerza modelo integrativo de sistemas biológicos
6	Boveri (1902)	Análisis del núcleo celular y herencia (n/a)	Estudio experimental clásico; calidad alta	Mitosis multipolar y su implicación genética	Fundamenta bases de genética celular
7	Briscoe &	Diseño de	Revisión	Gradientes	Importancia de

	Small (2015)	patrones embrionarios (modelos animales)	científica; calidad alta	morfogenéticos en desarrollo	gradientes en morfogénesis
8	Carus (1846)	Desarrollo del alma y noción de inconsciente (teórico)	Marco teórico; calidad conceptual	Relación entre biología y psicología	Aporta fundamentos históricos y conceptuales
9	Creighton et al. (2020)	Epigenética y memoria (humanos y animales, n=variable)	Revisión científica; calidad moderada	Epigenética en aprendizaje y envejecimiento	Destaca papel epigenético en memoria
10	Darwin (1859) [Reedición 2003]	Fundamentos de la evolución biológica (observación natural)	Teórico; calidad alta	Selección natural como motor evolutivo	Base para biología evolutiva
...	...	...	...	...	...
30	Westen (1999)	Procesos inconscientes y psicoanálisis (n/a)	Revisión teórica; calidad moderada	Reevaluación de Freud desde neurociencia	Integra psicoanálisis y neurociencia
